# Social Media Terms and Conditions and Informed Consent From Children: Ethical Analysis

**DOI:** 10.2196/22281

**Published:** 2021-04-22

**Authors:** Christophe Olivier Schneble, Maddalena Favaretto, Bernice Simonne Elger, David Martin Shaw

**Affiliations:** 1 Institute of Biomedical Ethics University of Basel Basel Switzerland

**Keywords:** social media, big data, ethics, children, health data, terms and conditions, trusted partnership, medical ethics, mobile phone

## Abstract

**Background:**

Terms and conditions define the relationship between social media companies and users. However, these legal agreements are long and written in a complex language. It remains questionable whether users understand the terms and conditions and are aware of the consequences of joining such a network. With children from a young age interacting with social media, companies are acquiring large amounts of data, resulting in longitudinal data sets that most researchers can only dream of. The use of social media by children is highly relevant to their mental and physical health for 2 reasons: their health can be adversely affected by social media and their data can be used to conduct health research.

**Objective:**

The aim of this paper is to offer an ethical analysis of how the most common social media apps and services inform users and obtain their consent regarding privacy and other issues and to discuss how lessons from research ethics can lead to trusted partnerships between users and social media companies. Our paper focuses on children, who represent a sensitive group among users of social media platforms.

**Methods:**

A thematic analysis of the terms and conditions of the 20 most popular social media platforms and the 2 predominant mobile phone ecosystems (Android and iOS) was conducted. The results of this analysis served as the basis for scoring these platforms.

**Results:**

The analysis showed that most platforms comply with the age requirements issued by legislators. However, the consent process during sign-up was not taken seriously. Terms and conditions are often too long and difficult to understand, especially for younger users. The same applies to age verification, which is not realized proactively but instead relies on other users who report underaged users.

**Conclusions:**

This study reveals that social media networks are still lacking in many respects regarding the adequate protection of children. Consent procedures are flawed because they are too complex, and in some cases, children can create social media accounts without sufficient age verification or parental oversight. Adopting measures based on key ethical principles will safeguard the health and well-being of children. This could mean standardizing the registration process in accordance with modern research ethics procedures: give users the key facts that they need in a format that can be read easily and quickly, rather than forcing them to wade through chapters of legal language that they cannot understand. Improving these processes would help safeguard the mental health of children and other social media users.

## Introduction

### Background

Social media companies have experienced tremendous growth during the last decade; however, they have largely neglected the issues of privacy and confidentiality. In addition to connecting people, social media apps (the companies) are also tremendous *data collectors*, gathering a wide range of information that spans from nonsensitive to highly sensitive data. Although many data might be nonsensitive in isolation, the combination of various types of data might subsequently allow insights into sensitive health issues [1]. In fact, many studies have used social media data to gain insights into the mental state of users [2,3]. Moreover, with children and young adults using social media apps from a young age, companies have acquired data over long time spans, which is similar to longitudinal data used in research. Keeping this in mind and knowing that predictive algorithms will become more accurate, it is of major importance to build governance and inform users about the use of their data to foster data protection. This is all the more important given the latest scandal surrounding Cambridge Analytica [4,5] and the sharing of data between Facebook and device manufacturers such as Apple and top-rated apps such as Spotify and Netflix [6]. These are prominent examples of misbehavior that illustrate the urgent need for a trusted partnership between users and social media companies.

Contractual law in the terms and conditions (also known as terms of services) and privacy policies define how privacy, confidentiality, and data sharing are handled. They are the predominant legal and contractual mechanisms that define the relationship between users and social media companies. These mechanisms are subject to various national and international regulations. The General Data Protection Regulation (GDPR) of the European Union (EU) [7] sets boundaries concerning the processing of data. In the United States, the Children’s Online Privacy Protection Act (COPPA) [8] and the fair information principles issued by the Federal Trade Commission [9] are the 2 predominant regulations.

When signing up for such a service, users consent by reading or at least scrolling through the terms of service and by clicking the *agree* button. However, these terms and conditions are often long and written in a complex legal language. Thus, it remains questionable whether users—particularly children and young adults—truly understand the terms and conditions and are aware of the consequences of joining a network. Most of the platforms offer their service for free but require users to accept the preset *package* of conditions with limited privacy choices to permit access to their services.

Social media apps are ubiquitous in today’s world and have changed the way we communicate, share, and interact with each other daily. They are also omnipresent in the lives of young people, and it is estimated that 1 in 3 of all internet users is under the age of 18 years [10,11]. A recent study by the UK Children’s Commissioner has shown that a significant number of children access social media through their parents’ accounts, whereas most adolescents (71% in the United States and 85% in Europe) have one or more social media accounts or identities [12]. When children access social media through their parents’ accounts, parents might feel that they have control over their children’s media use. This is problematic for 2 reasons: first, parents will not be able to control every click, and second, as the UK Children’s Commissioner points out, children might be presented with explicit adult content of which their parents remain unaware.

Letting children use parents’ accounts also bypasses the age requirements imposed by social media companies. In their terms of service, social media apps and services defined the minimum age at which adolescents or children can use the app or service without obtaining parental consent. With regard to age requirements, the law plays an important role by setting boundaries for protecting children’s privacy, data sharing, and profiling. In the United States, COPPA defines 13 years as the minimum age to join such communities. Before that age, explicit parental consent is needed to sign up. The EU has recently introduced the GDPR, in which Article 8 defines the necessity of parental consent for all youths aged below 16 years in situations where information society services are offered directly to them. However, the member states are free to choose and adopt their own particular regulation within the age range of 13-16 years. Some countries, such as the United Kingdom, have opted for an age of 13 years, whereas others such as Germany have set the boundary at 16 years [10]. The GDPR would thus not prohibit the use of such services before the minimum age requiring children’s self-consent but would instead require parental consent to access these services and process the personal data of children, as defined in the GDPR. Most of the companies however set their minimum age requirements at the age imposed by national law, as shown in our results.

However, the efficacy of such age regulations remains to be questionable as the primary research strands in children’s digital rights show that children and parents feel social pressure to join such communities [12] and thus might lie about their age when joining social media services [13]. Doing so is easy because normally, signing up relies only on the honesty of the user.

### Objectives

This paper provides an ethical analysis of the most popular social media platforms and services used by children and adolescents (in the EU and the United States). It focuses on age requirements, how information about the platform is presented, how consent is obtained, how (and if) age verification is implemented, whether resources are provided to educate parents or children, and if there are community guidelines. It then discusses the emerging issues and the predominant regulations of our target countries and illustrates how experiences from research ethics could be used to develop a trusted relationship between users and companies, facilitating the *ethical* functioning of social media networks.

## Methods

We conducted a thematic analysis [14] of the terms and conditions of the 20 most popular social media platforms in 2019 [15] and the 2 predominant mobile phone ecosystems, Android and iOS. Within this sample of 20 platforms, we excluded all apps and social networks targeting only Chinese-speaking users (because of a lack of terms and conditions in English; WeChat, QQ, QZone, and Sina Weibo), discussion websites (Reddit), and those targeting only adults (LinkedIn or Viber), resulting in 10 platforms relevant to children. The terms and conditions were read in depth, emerging topics of ethical interest were identified, and categories for further in-depth analysis were created. The categories identified were the minimum age to join, how the consent process was handled, the age verification process, the presence of parental portals (educating parents on the use of the respective platforms), and the possibility of requesting account deletion in the cases of underaged users. Note that most of the platforms are available either as web apps or as smartphone apps. The results of this in-depth analysis are summarized in Table 1, and the apps are scored according to the criteria in Table 2. As most of the apps are available on smartphones, we also decided to include the quasi-standard platforms such as Android and Google, as they have a gatekeeping function (in terms of age) to allow children to access those networks.

**Table 1:** Overview of the most popular social media apps.

Platform or app		Active users (in millions)	Provider	Predominant content	Viewable without signing in	Minimum age (years)	Age verification	Possibility to request deletion of the account	Parental consent	Parent portal or community guidelines
**Social media**										
	Facebook	2234	Facebook Inc	Video or text or images or social messaging	Yes	13	Verification of official document when account is locked	Yes (form)	Consent by user	Yes
	YouTube	1900	Google	Video creation	Yes	13 (≥14/≥16)^a^	Background check or verification of official document or credit card verification when locked	Yes	Consent by user or parents if below 13 years	Yes
	WhatsApp	1500	WhatsApp Inc (Facebook Inc)	Social messaging (video or text or music)	No	13	By SMS messaging	No	Consent by user	No
	Instagram	1000	Facebook Inc	Images or video	Yes	13 (16)^a^	Verification of ID when locked	Yes (form)	Consent by user	Yes
	TikTok	500	Beijing Bytedance Technology	Music or images	No	13 (14)^a^	No	Yes (mail)	Yes (for certain countries)	No
	Twitter	335	Twitter Inc	Text	Yes	No	Yes for sensitive posts	Yes	Consent by user	No
	Skype	300	Microsoft Corporation	Social messaging	No	No	No	No	Consent by user	No
	Snapchat	291	Snap Inc	Video or photo posting	No	13	By peer or birthday can be changed only a limited number of times	Yes (mail)	Consent by user	Yes
	Pinterest	250	13	Images	Yes	13	By peer	Yes (form)	Consent by parents if underaged use	Yes
	LINE	203	LINE Corporation	Social messaging	No	No	No	No	No	No
**Ecosystems**										
	iOS (Apple ID)	N/A^b^	Apple	Apps	N/A	13	Yes (Credit card or SMS)	Yes	Consent by parents if underaged users	Yes
	Android Play Store	N/A	Google	Apps	N/A	13 (≥14/≥16)^a^	Back check or verification of official document or credit card verification	Yes	Consent by parents if underaged users	Yes

^a^On the basis of the country, the companies have adopted a different minimum age.

^b^N/A: not applicable.

**Table 2:** Scoring the most popular social media apps.

Platform or app	Minimum age or age verification	Parental consent	Possibility to request deletion of the account	Parent portal or community guidelines	Total score
Facebook	Age restriction and implemented age verification present	Consent by user	Yes	Parent portal present	3
YouTube	Age restriction and implemented age verification present	Consent by parents	Yes	Parent portal present	4
WhatsApp	Age restriction and implemented age verification present	Consent by parents	No	No parent portal	2
Instagram	Age restriction and implemented age verification present	Consent by user	Yes	Parent portal present	3
TikTok	No age restriction or no age verification present	Consent by parents	No	No parent portal	1
Twitter	No age restriction or no age verification present	Consent by user	Yes	No parent portal	1
Skype	Age restriction and implemented age verification present	Consent by parents	No	No parent portal	2
Snapchat	Age restriction and implemented age verification present	Consent by user	Yes	Parent portal present	3
Pinterest	Age restriction and implemented age verification present	Consent by parents	Yes	Parent portal present	4
LINE	Age restriction and implemented age verification present	Consent by user	No	No parent portal	1

## Results

The results of our analysis will be discussed thematically, in turn, after presenting the results of our scoring mechanism.

### Scoring System

On the basis of the data in Table 1, our scoring system (Table 2) awards each platform a possible score of 1 (+) or 0 (none) across the 5 different categories used in our analysis. The criteria are presented in Table 3. The category for minimum age and age verification is cumulative. One point will be awarded only if both criteria are met, because we believe this fulfills the gatekeeper function. Studies suggest that children are often happy to lie about their age and that parents even encourage their children to sign up [13,16]; thus, the efficacy of a minimum age requirement in the absence of verification remains ethically questionable.

**Table 3:** Constraints of the scoring system.

Topic	Criteria for point	Criteria for no point
Minimum age or age verification	Age restriction and implemented age verification present	No age restriction or no age verification present
Possibility to request deletion	Yes	No
(Parental) consent process	Consent by parents	Consent by user
Parent portal	Parent portal present	No parent portal

### Age Requirements and Age Verification

Table 1 shows that all companies except LINE have adopted a minimum age of 13 years for the use of their services. However, the Apple and Google (Android) ecosystems offer the possibility of using their various services at a younger age with parental consent. Google achieves this by integrating the child’s account into the so-called *Family Link* [17], a platform to group and administrate family member accounts; the same applies to Apple, which has also set up an infrastructure to manage family accounts. Most service providers rely on other users reporting underage use and offer either a mailing address or a form as the only way of contact when requesting the deletion of an account created by underage children. A more sophisticated method has been adopted by Google, where a background check is performed by verifying the age entered in any one of its services whenever the user uses another service that is part of its ecosystem. Once an account is locked, Instagram and Facebook request a copy of an official document (ID card or passport) to unlock it. Android, iOS, and YouTube adopt another way of handling this issue, where the check is performed against a valid credit card, resulting in a parent giving de facto consent. In contrast, Snapchat allows users to change their date of birth only a certain number of times [18].

### Consent Process

Upon registration, the user was asked to accept the terms and conditions. In most cases, the user agrees to the terms and conditions by checking a checkbox and subsequently clicking the *register* button or even by only clicking the *register* button (Facebook and Instagram).

Sometimes, the link to the terms and conditions is in a smaller font (see Table S2 in Multimedia Appendix 1 for an overview) so that it is hardly identifiable (Snapchat). On Instagram and Facebook, it is highlighted in bold font. Although the Article 29 Working Party (an independent European advisory body on data protection and privacy created by the EU) offers some recommendations on the consent process [19], we were not able to identify a standard presentation form or standard procedure in presenting terms and conditions. Most forms show their terms and conditions only in continuous text, whereas others have adopted a question and answer form (eg, Facebook, Instagram, and Pinterest). Pinterest is the only platform that provides a simplified version in addition to the full version of its terms (Textbox 1).

**Full text versus simplified terms and conditions (Pinterest).**

**Full text**

You grant Pinterest and our users a non-exclusive, royalty-free, transferable, sublicensable, worldwide license to use, store, display, reproduce, save, modify, create derivative works, perform, and distribute your User Content on Pinterest solely for the purposes of operating, developing, providing, and using Pinterest. Nothing in these Terms restricts other legal rights Pinterest may have to User Content, for example under other licenses. We reserve the right to remove or modify User Content or change the way it’s used in Pinterest, for any reason. This includes User Content that we believe violates these Terms, our Community Guidelines, or any other policies.

**Simplified version**

If you post your content on Pinterest, we can show it to people and others can save it. Don't post porn or spam or be a jerk to other people on Pinterest.

### Parent Portals or Community Guidelines

Almost every platform (except social messaging platforms) offers a parent’s portal or community guidelines. This ranges from simply linking to interesting articles (Snapchat) to providing an information center (Instagram and Facebook) to video sequences (Facebook) on problematic behavior along with short sequences showing a safe way to use the service.

## Discussions

### Principal Findings

On the basis of our scoring system (Table 2), most providers scored 3 out of 4 points. However, one-third of the service providers achieved poor results. This shows that the regulations that service providers comply with, either by themselves or by law, offer at least some protection for users. However, TikTok, Twitter, and LINE only scored 1 point and only 2 companies achieved the maximum score (Pinterest and YouTube).

In the following section, we will therefore discuss the categories presented in Table 1 and suggest possible improvements within the framework of the 4 guiding ethical principles.

### Minimum Age to Sign Up for a Service

Our analysis reveals that most apps have adopted the minimum age of 13 years for children to sign up to use their services. This complies with the US COPPA and GDPR. In contrast with the COPPA, the GDPR provides a minimum age requirement ranging from 13 to 16 years for children to register for a service. Owing to the GDPR’s extraterritorial force (as mentioned in Article 3 of the GDPR), other states and companies outside the EU have to comply with EU standards when targeting users (and children) in an EU member state.

Strongly intertwined with the definition of the minimum age is the issue of age verification. As Table 1 shows, the issue of age verification is currently not taken seriously by companies, and an age requirement is largely useless in the absence of verification. Therefore, we argue that a robust age verification process needs to be adopted by service providers in the coming years. However, establishing such mechanisms needs to be implemented in a way that complies with privacy and the principles of data minimization [19]. The survey mentioned earlier [13] has shown that some children lie about their age and the ease of registering for a social media service (requiring only a few minutes) does not constitute a barrier.

Currently, some providers request verification by email or phone by sending the user a short message during the registration process (the standard procedure for setting up a WhatsApp account). The latter provides an additional security layer as cell phone companies have a minimum age for issuing a contract; when a child has a cell phone, the parents have at least agreed to the use of such a device and thus are aware that the child might sign up for such a service, even if they are potentially unaware of the services that the child subsequently signs up for. However, this might be a problem in countries where pay-as-you-go phones require no identification, either by age or by verification with an official ID card or social security card. Furthermore, implementing an age verification process by requesting verification through a text message could be seen as discriminating against children who do not possess a cell phone at all and, thus, solely have to rely on a parent to register.

Other providers delegate *age verification* to their users by setting up forms where one can report underage use. However, this method does not guarantee age verification and, in the absence of other measures, it suggests that the service provider is neither serious nor proactively interested in complying with the minimum age requirement.

Today's technologies could make it possible to approach the minimum age to check more proactively. For example, artificial intelligence could enable the use of techniques such as image classification algorithms or natural language processing to detect underage children by analyzing their physical face properties (such as the Amazon *recognition application programming interface* [20]) or using written language with neurolinguistic programming for processing natural language. We are fully aware that the use of such technologies can lead to other ethical and legal concerns. Although these concerns are too complex to address in depth in this paper, we discuss them briefly in the following section.

Article 9 of the GDPR places biometric data in a special category: processing is prohibited unless special circumstances are met. However, notably Article 9 [7] of the GDPR permits each EU member country to introduce certain derogations with respect to restrictions on processing biometric data (*member states may maintain or introduce further conditions, including limitations*). For instance, the Netherlands has provided an opt-out option for biometric data if necessary, for authentication or security purposes, and Croatia’s new data protection law exempts surveillance security systems [21]. In the United States, no federal law regulates the collection of biometric data. However, 3 states—Illinois, Washington, and Texas—have implemented regulations on biometric data [21]. On the ethical side, the introduction of such technologies to tackle the issue of age verification is also potentially problematic, as appropriate consent must be obtained from the user, who should also have a full overview where the biometric data are being used, as these types of data represent special categories that are harmful when misused. Thus, the use of such technologies should follow clear ethical guidelines. For example, such technologies should not be used to collect more information about users and data than is necessary, and they should always be used for a specific purpose. This is also because an increasing number of predictive analyses are possible [2,22] from simple social media data.

### Obtaining Consent

Obtaining valid user consent (and in the case of children, parental consent) is one of the 6 lawful bases to process personal data, as listed in Article 6 of the GDPR. Generally, as consent is a tool that gives users *data subjects* control over whether personal data concerning them will be processed [19], to do so, valid consent has to meet certain criteria; it must be freely given, be specific, and be informed and include an unambiguous indication of the data subject’s wishes. How consent is presented to the user, whether it is written or presented pictorially or in short video sequences, is up to the controller (company). This means that harmonization is not currently envisaged. However, the Article 29 Working Party (an advisory board of the EU on data protection issues) does lay out how data subjects (users) should provide consent. Obtaining consent by simply scrolling down and ticking a checkbox is not seen as appropriate from an ethical standpoint, although it might be sufficient from a policy perspective. Thus, the Working Party provides 2 examples of how a valid mechanism could look (outlined in Textbox 2), which is not currently met by any of the services that are subject to our investigation. As shown in our analysis, users are presented with written information on their rights and rights of companies on topics such as data protection, community rules, and minimum age. A further issue is that some of the services only provide a checkbox to tick or, in the worst case, only a button to register where the terms and conditions are not displayed during the account’s creation unless the user clicks the link. This fosters a *click and forget* mentality and is far from providing a sustainable and respectful partnership between service providers and users. Often, the link to the terms and conditions is presented in smaller fonts and stands in contrast with the large textboxes filled during the registration process, as shown in the examples in Table S2 in Multimedia Appendix 1.

**Example of how to obtain consent (examples of the Article 29 Working Party).**

**Appropriate way**

Swiping a bar on a screen, waiving in front of a smart camera, turning a smartphone around clockwise, or in a figure-eight motion may be options to indicate agreement, as long as clear information is provided, and it is clear that the motion in question signifies agreement to a specific request (e.g., if you swipe this bar to the left, you agree to the use of information X for purpose Y. Repeat the motion to confirm). The controller must be able to demonstrate that consent was obtained this way, and data subjects must be able to withdraw consent as easily as it was given.

**Inappropriate way**

Scrolling down or swiping through a website will not satisfy the requirement of a clear and affirmative action. This is because the alert that continuing to scroll will constitute consent may be difficult to distinguish and/or maybe missed when a data subject is quickly scrolling through large amounts of text and such an action is not sufficiently unambiguous.

A special category for obtaining consent is imposed for children below the age of legal maturity in their respective countries. In such cases, the GDPR and COPPA require approval from the parent or guardian. This has several positive and negative aspects. On the one hand, this regulation places the burden on the parents to protect children from potential harm, which could, in turn, be built by safeguarding mechanisms of the platforms. On the other hand, overrestrictive consent processes could be a driver of inequality, as strict parents could hinder beneficial usage. A complex consent process (such as using the parents’ credit card or facial recognition) is always associated with more data being collected not only from the child but also from the parent. Thus, balancing data minimization against sufficient safeguards plays an important role in designing an ethical consent process.

Emphasizing consent is important; however, other scholars have argued that solely focusing on this aspect and implying parental consent is not enough. By making data protection impact assessment mandatory (as required by the GDPR), risks can be already identified at an earlier stage [22]. Combining these 2 approaches for making the terms and conditions more readable and fostering data protection impact assessments would help to protect children’s rights.

### Educating Users and Parents

As the report of the UK Children’s Commissioner [12] has shown, the safe use of social media depends on building awareness and educating children about its use and fostering digital literacy. Parents and teachers play an important role. Most of the apps we analyzed offered parents websites where the companies either provided links to useful literature (the simplest way to deal with that issue) or by providing short YouTube sequences to inform children and parents about potential harm and the security measures to take when using social media.

Given the importance of educating parents and teens [12], we suggest that future legislation should mandate the implementation of such parental portals. From an ethical point of view, it would be good to encourage companies to spend a reasonable amount of their revenue in educating parents and children about the potential harm resulting from the use of their services. A good example is provided in the Facebook Help Center, which offers short YouTube sequences and quizzes on the topics of data protection and possible harm.

### Social Pressure

Social media apps have become ubiquitous among children and adolescents. It has become difficult to refuse to be part of such networks, because of both social pressure and an increasing number of institutions (such as schools) requiring such channels, resulting in social pressure to use these services for communication, regardless of whether parents regard the use of these services to be appropriate for their children. This could also be seen as a loss of autonomy concerning the freedom to decide whether and when to join. We can imagine a scenario in which children who want to participate in social media life are pressured to lie about their age on the internet by fellow schoolmates or friends because this peer group’s main vehicle of social interaction is heavily mediated by online- messaging and social media, for example, children need to be on WhatsApp to be able to meet with others because all of the peer meetings are communicated that way. It is also possible that parents could incentivize their offspring to engage in online misconduct as they want their children to use online messaging services (eg, WhatsApp) out of convenience or for monitoring purposes. These phenomena can create new social inequalities. In fact, in its 2017 report, UNICEF (United Nations International Children's Emergency Fund) warned of the formation of a significant digital divide [23], highlighting the gap between children who can connect and subsequently sign up for social media networks. This divide could be the result of either having more permissive parents who agree to the use of such services or because the child is wealthy enough to purchase a pay-as-you-go phone with data to access social media services secretly. Conversely, children who are left out of social media because their parents are more law-abiding or controlling or because their socioeconomically disadvantaged background makes personal phones unaffordable or are forced to share their parents’ devices. Children in the latter group feel left out of their friends’ social lives and end up being ostracized by their peers or even bullied.

With the introduction of the GDPR and the adjustment of the minimum age to 16 years in certain countries, it is expected that the topic of social pressure will defuse itself at least on an institutional level because institutions must adhere to this requirement. However, social media companies’ adhesion to the GDPR age requirement could, on the other hand, worsen social pressure for children as the gap between the legal age at which it is possible to join social media and children’s actual social practices differs [24]. In medical care, children can give consent for themselves below the legal age of maturity; however, this exception does not apply in the case of compliance with GDPR.

### Research Ethics as a Model for a Trust-Based Partnership

**Figure 1:**
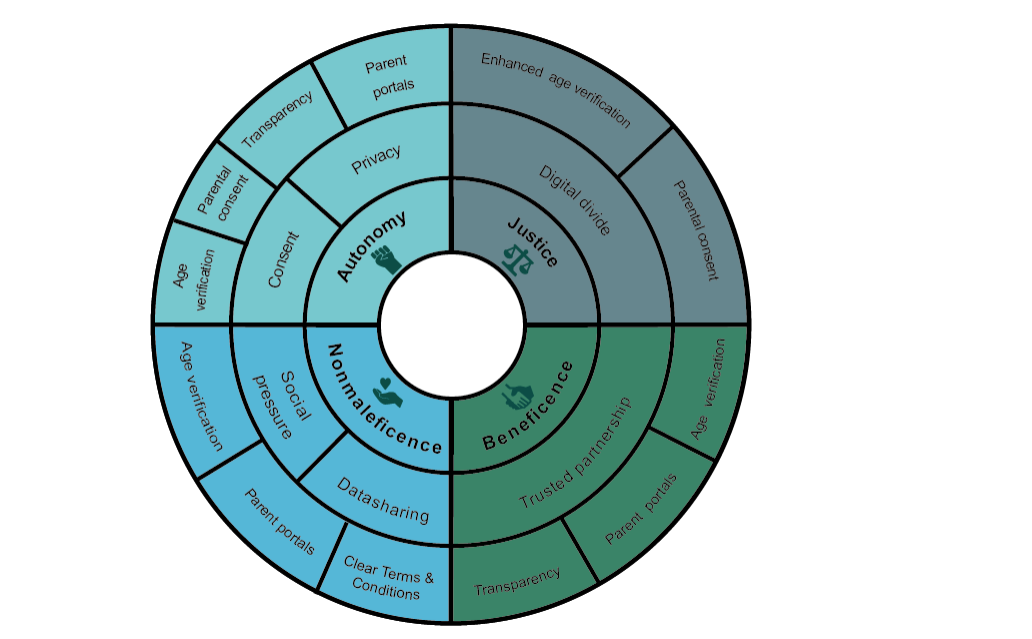
Mapping the four ethical biomedical principles of the use of social media to issues arising from the use of social media and links them to possible fields of actions. (Enlarged age verification: Using sophisticated mechanisms such as credit card charges could foster digital divide; Parental consent: Parents might prevent kids joining resulting in negative consequences for them).

Similar to social media today, biomedical research used to have a bad reputation in terms of involving participants. People were included in medical studies without their consent, and their data were shared without their knowledge. To prevent such unethical practices, 4 main ethical principles have become fundamental to research ethics and biomedical ethics more widely: respect for autonomy, nonmaleficence, beneficence, and justice. In the context of social media, all of these principles are relevant; however, this is particularly true of respect for autonomy and nonmaleficence. Figure 1 illustrates how social media can innovate to ensure age verification, valid consent, and other aspects to make sure that these key ethical principles are respected. Fundamentally, it is an ethical imperative to ensure that children are of suitable age and understand the risks of social media to reduce the risk of harm to their emotional well-being and mental health: evidence suggests that social media can have substantial impacts in the areas of *self-esteem and well-being, with issues related to cyberbullying and Facebook Depression* [25].

In research ethics, the informed consent process plays a crucial role and contributes to a trusted partnership between subjects and researchers. When approached about the possibility of involvement in a clinical study (and increasingly for interviews or survey participation), potential participants are given all relevant information and time to digest and consider it before signing an informed consent form. In the past, the information provided to participants often ran to over 100 pages, thus raising the same concerns about accessibility and comprehensibility as social media terms and conditions. In recent years, however, there has been a move toward making such information much more patient- and participant-friendly, with, for example, the UK Human Research Authority supporting the use of simple information sheets in a question and answer format running to a maximum of 5-10 pages. This practice focus on communicating relevant information about risks and harms in a concise and comprehensible format could also serve as a model for building trusted relationships between social media users and companies. The problem with using terms and conditions as an information sheet is that such policies are essentially legal documents and written in dense legal language. Disentangling lengthy legal texts from the salient information required to provide informed consent is essential for social media companies. However, today’s relationships are still unbalanced from the very beginning, with users required to sign up with a simple click after having to read information that is only presented in written form and complex language. This means that many users remain to be unaware of exactly what they are signing up for. Moving toward some sort of pictorial consent system would be a much more appropriate approach to informing both children and adults about the risks of social media use. This debate is not new in the legal context; Brunschwig [26] was one of the first to show how contractual law can be exemplified with comics fostering a better understanding of otherwise complex matters. Several scholars have been working on this topic, proposing *nutrition label–like* terms and conditions [27] and grid-based terms and conditions [28]. Such pictorial forms of consent are best practices in research ethics settings, especially with sensitive study participants or those with low literacy levels. There might be some implementation issues with such solutions. Nevertheless, when we are speaking about children—*a sensitive group*—such terms and conditions are a much better means of informing users about potential harm. This is not a purely theoretical discussion and approach, as Apple recently presented *nutrition labels for their App Store* [29].

Another possible solution, and a step in the right direction, is the simplified text-based rules for several social media apps developed by the UK Children's Commissioner [30]. Research ethics also requires that data can typically only be shared and processed with the consent of the persons concerned. However, recent social media scandals [4,31] have shown that some social media companies have neglected this issue, which must also be addressed more clearly in terms and conditions. Another essential aspect of research ethics is the right to withdraw consent and the possibility of deleting data (or an account if research takes place via the internet) by the user. However, for underaged users (with respect to the minimum age required by the companies), it should also be possible for parents to delete an account without going through a complicated process. This could be done, for example, by specifying a parental contact when registering the account. Finally, research ethics also address the potential risks in participating in a study. Most companies in our sample address possible harms of using their services in their parent portals and community guidelines.

### Conclusions

Our analysis reveals that social media networks are still lacking in many respects with regard to adequate protection for children. Consent procedures are flawed because they are too complex, and in some cases, children can create social media accounts without sufficient age verification or parental oversight. Given the high risks of inappropriate content being shared and the targeting of children with specific advertisements, social media companies must improve their procedures to protect not only children but also all users. This can be achieved by standardizing the registration process in accordance with modern research ethics procedures described earlier: give users the key facts that they need in a format that can be read easily and quickly, rather than forcing them to wade through chapters of legal language that they cannot understand. Disentangling the practical information that users need from the complex legal language would also have the benefit of facilitating standardization; regardless of the jurisdictions, the language for consent documents should be simple and straightforward. In addition, in some cases, using pictorial versions of the terms and conditions would surely leverage the efficacy of today’s mostly unread versions. The vast majority of social media users have given only uninformed consent; however, the *click, consent, and forget at your peril* model must be relegated to history in favor of a more transparent and ethical system. The standardization of terms and conditions is only possible if an effective political intervention is implemented. Recent developments and discussions about monopolistic large social media companies in the US Congress are a step toward harmonization. Furthermore, the role model function of the GDPR as a quasi-standard for new data protection regulations will eventually simplify standardization. Adopting measures based on key ethical principles will safeguard children’s health and well-being and those of other social media users.

## Appendix

Multimedia Appendix 1 Links and presentation of terms and conditions.

## Abbreviations

COPPA: Children's Online Privacy Protection Act
EU: European Union
GDPR: General Data Protection Regulation

## References

[1] Schneble C, Elger B, Shaw D (2020). All Our Data Will Be Health Data One Day: The Need for Universal Data Protection and Comprehensive Consent. J Med Internet Res.

[2] Reece A, Danforth C (2017). Instagram photos reveal predictive markers of depression. EPJ Data Sci.

[3] Eichstaedt JC, Smith RJ, Merchant RM, Ungar LH, Crutchley P, Preoţiuc-Pietro D, Asch DA, Schwartz HA (2018). Facebook language predicts depression in medical records. Proc Natl Acad Sci U S A.

[4] Schneble CO, Elger BS, Shaw D (2018). The Cambridge Analytica affair and Internet-mediated research. EMBO Rep.

[5] (2018). The Cambridge Analytica Files. The Guardian.

[6] Alex H (2018). Facebook discussed cashing in on user data, emails suggest. The Guardian Internet.

[7] The European Parliment and The Council of The European Union EPCOTEU (2016). General Data Protection Regulation. Official Journal of the European Union.

[8] The 105th United States Congress (2000). Children's Online Privacy Protection Act, USA.

[9] Federal Trade Commission (1998). Privacy Online: A Report to Congress. https://www.ftc.gov/sites/default/files/documents/reports/privacy-online-report-congress/priv-23a.pdf.

[10] Milkaite I, Lievens E (2019). Children's Rights to Privacy and Data Protection Around the World: Challenges in the Digital Realm. Eur J Law Technol Internet.

[11] Brian Ed, Little C (2017). State of the Worlds Children - Children in a Digital World Internet. UNICEF.

[12] Butterfill R, Charlotte M-P, Powell H, Nettleton O, Kriszner M (2018). Life in Likes: Children's Commissioner Report into Social Media use in among 8-12 Year Olds Internet. UK Children's Commissioner.

[13] Boyd D, Hargittai E, Schultz J, Palfrey J (2011). Why parents help their children lie to Facebook about age: Unintended consequences of the “Children’s Online Privacy Protection Act”. First Monday.

[14] Braun V, Clarke V (2006). Using thematic analysis in psychology. Qualitative Research in Psychology.

[15] Most famous social network sites worldwide as of October 2018, ranked by number of active users (in millions) Internet. Statista - The Statistics Portal.

[16] (2019). Why children spend time online Internet. British Office of Communications.

[17] Google Familiy Link Internet. Google Inc.

[18] (2018). Snapchat Support Internet. Snap Inc.

[19] (2018). Guidelines on Consent under Regulation 2016/679 Internet. Article 29 Working Party.

[20] (2018). Amazon Rekognition. Amazon.

[21] Whitener M, Aragon R How should we regulate facial-recognition technology?. International Association of Privacy Professionals.

[22] van der Hof S, Lievens E (2018). The Importance of Privacy by Design and Data Protection Impact Assessments in Strengthening Protection of Children's Personal Data Under the GDPR Internet. Communication Law.

[23] UNICEF (2017). The State of the World's Children. UNICEF.

[24] Livingstone S (2018). Children: A Special Case for Privacy. InterMedia.

[25] Richards D, Caldwell PH, Go H (2015). Impact of social media on the health of children and young people. J Paediatr Child Health.

[26] Brunschwig C (2001). Visualisierung von Rechtsnormen - Legal Design. Zürcher Studien zur Rechtsgeschichte.

[27] Kelley P, Bresee J, Cranor L, Reeder R (2009). A Nutrion Label for privacy. Proceedings of the 5th Symposium on Usable Privacy and Security - SOUPS 09 Internet.

[28] Reeder R, Kelley P, McDonald A, Cranor L (2008). A user study of the expandable grid applied to P3P privacy policy visualization. Proceedings of the 7th ACM workshop on Privacy in the electronic society.

[29] Morse J Apple's privacy-focused nutrition labels for apps are only a start. Mashable.

[30] (2018). Simplified Social Media Terms and Conditions for Facebook, Instagram, Snapchat, YouTube and WhatsApp Internet. UK Children's Commissioner.

[31] Schneble CO, Elger BS, Shaw DM (2020). Google's Project Nightingale highlights the necessity of data science ethics review. EMBO Mol Med.

