# Supplementary material for: Social Media Terms and Conditions and Informed Consent From Children: Ethical Analysis
Source: JMIR Pediatr Parent. 2021 Apr 22;4(2):e22281. doi: 10.2196/22281 (PMC8103294; doi:10.2196/22281)
Supplement: Multimedia Appendix 1 [file pediatrics_v4i2e22281_app1.docx]

## Appendix

Table 1. Link to the Terms and Conditions

| Platform/App | Link to the Terms and Conditions |
| --- | --- |
| Social Media |  |
| Facebook | https://web.facebook.com/legal/terms/update |
| YouTube | https://www.youtube.com/t/terms |
| WhatsApp | https://www.whatsapp.com/legal/?eea=1#terms-of-service |
| Instagram | https://help.instagram.com/581066165581870 |
| Tik Tok | https://www.tiktok.com/i18n/terms/ |
| Twitter | https://twitter.com/en/tos |
| Skype | https://www.microsoft.com/en/servicesagreement/ |
| Snapchat | https://www.snap.com/en-US/terms/ |
| Pinterest | https://policy.pinterest.com/en/terms-of-service |
| LINE | https://terms.line.me/line_terms/ |
| Ecosystems |  |
| IOS (Apple ID) | https://www.apple.com/legal/internet-services/itunes/us/terms.html |
| Android Play Store | https://play.google.com/about/play-terms/index.html |

Table 2. How are the Terms and Conditions presented?

| Facebook | | Instagram | | Snapchat |
| --- | --- | --- | --- | --- |
| 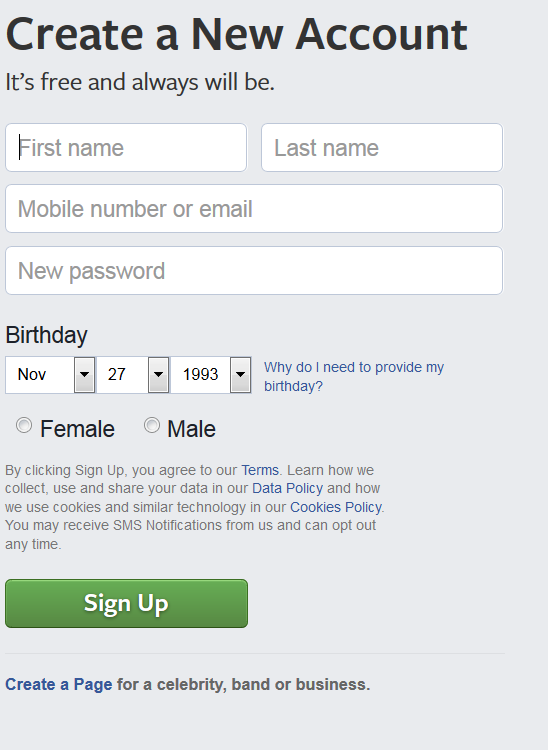 | | 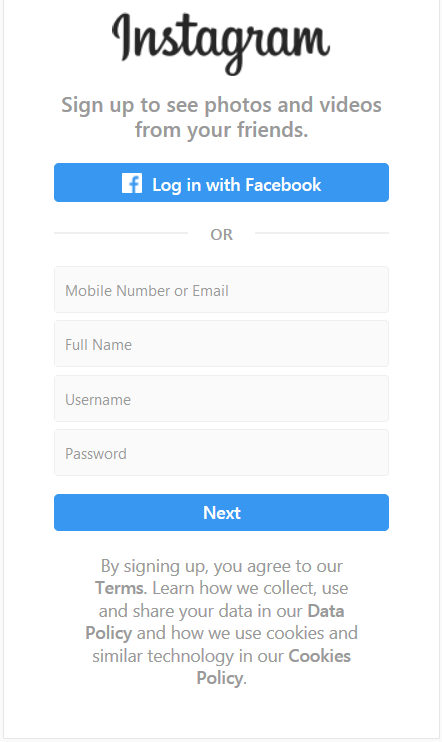 | | 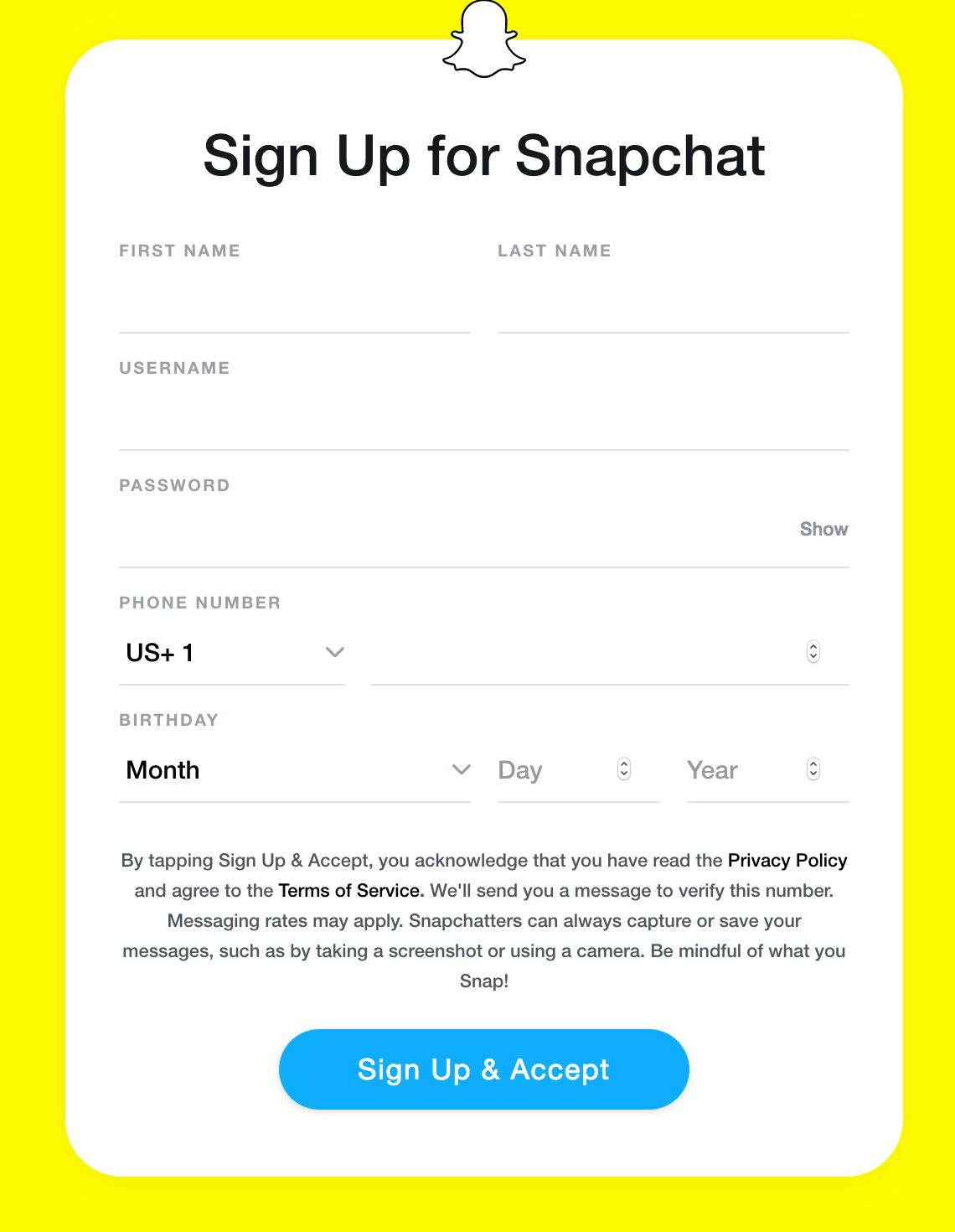 |
| YouTube | Tik Tok | | WhatsApp | |
| 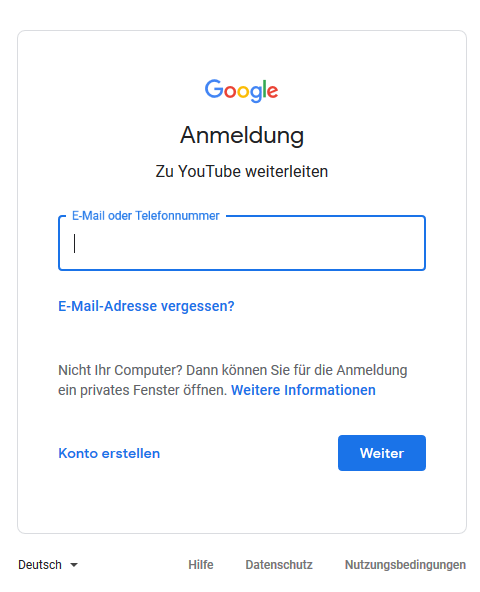 | 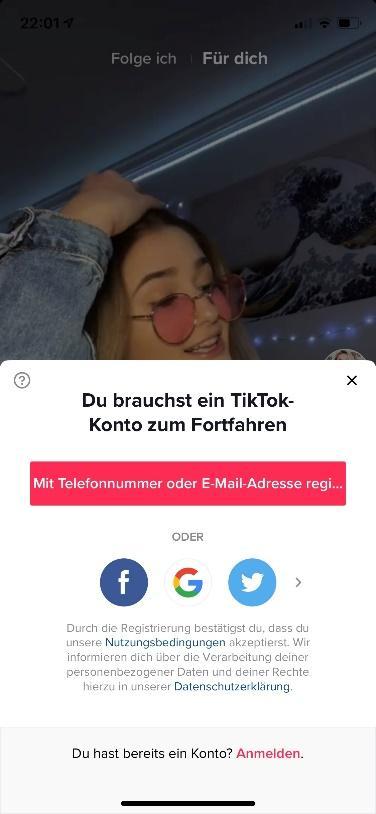 | | 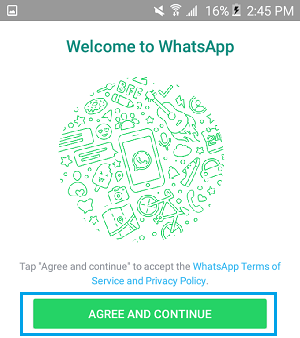 | |

| Pinterest | Twitter | | Line | |
| --- | --- | --- | --- | --- |
| 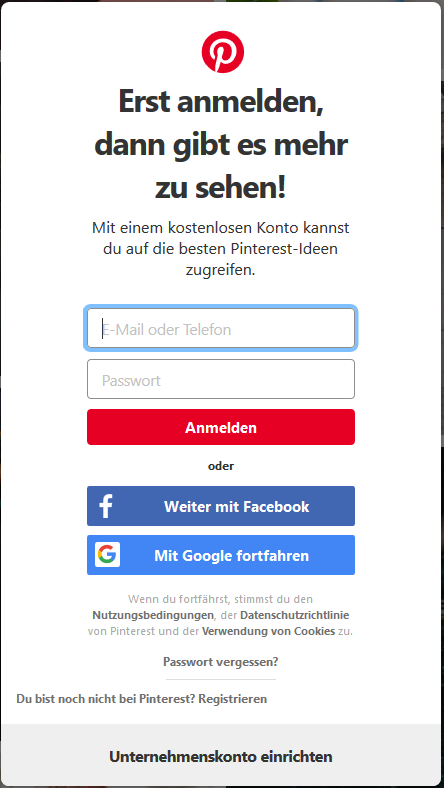 | 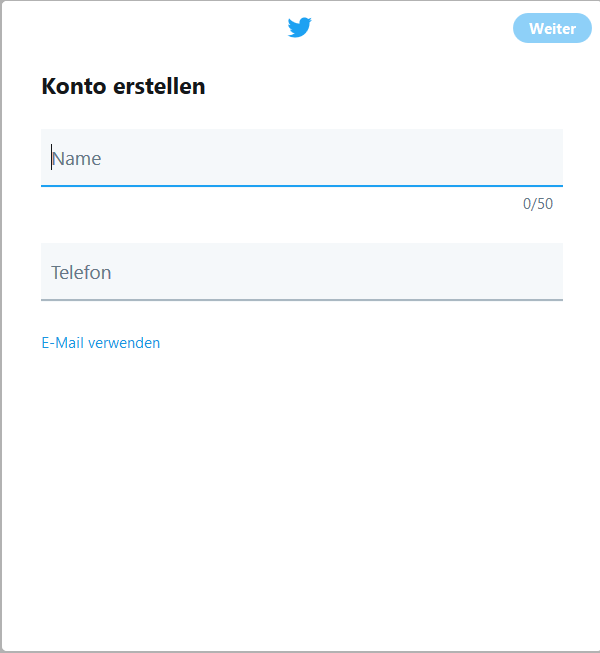 | | 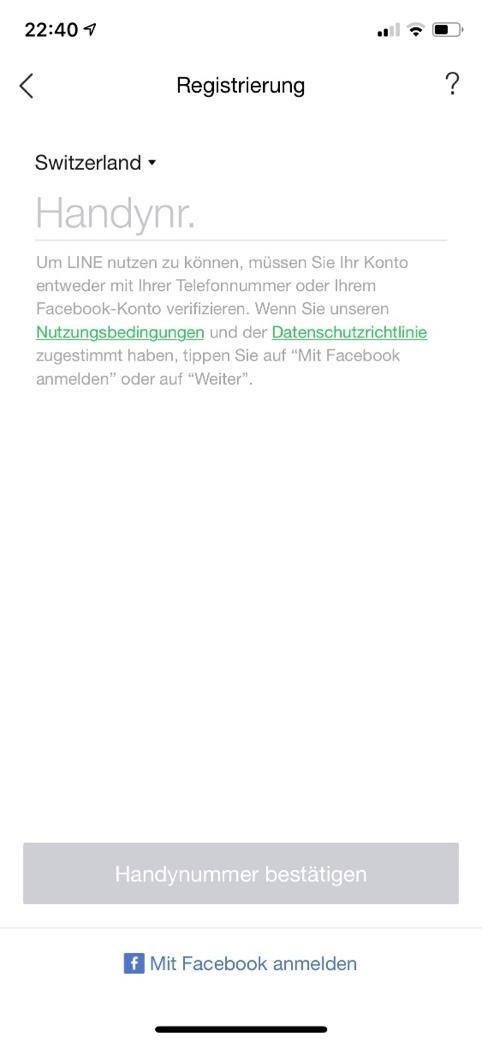 | |
| Skype | |  | |  |
| 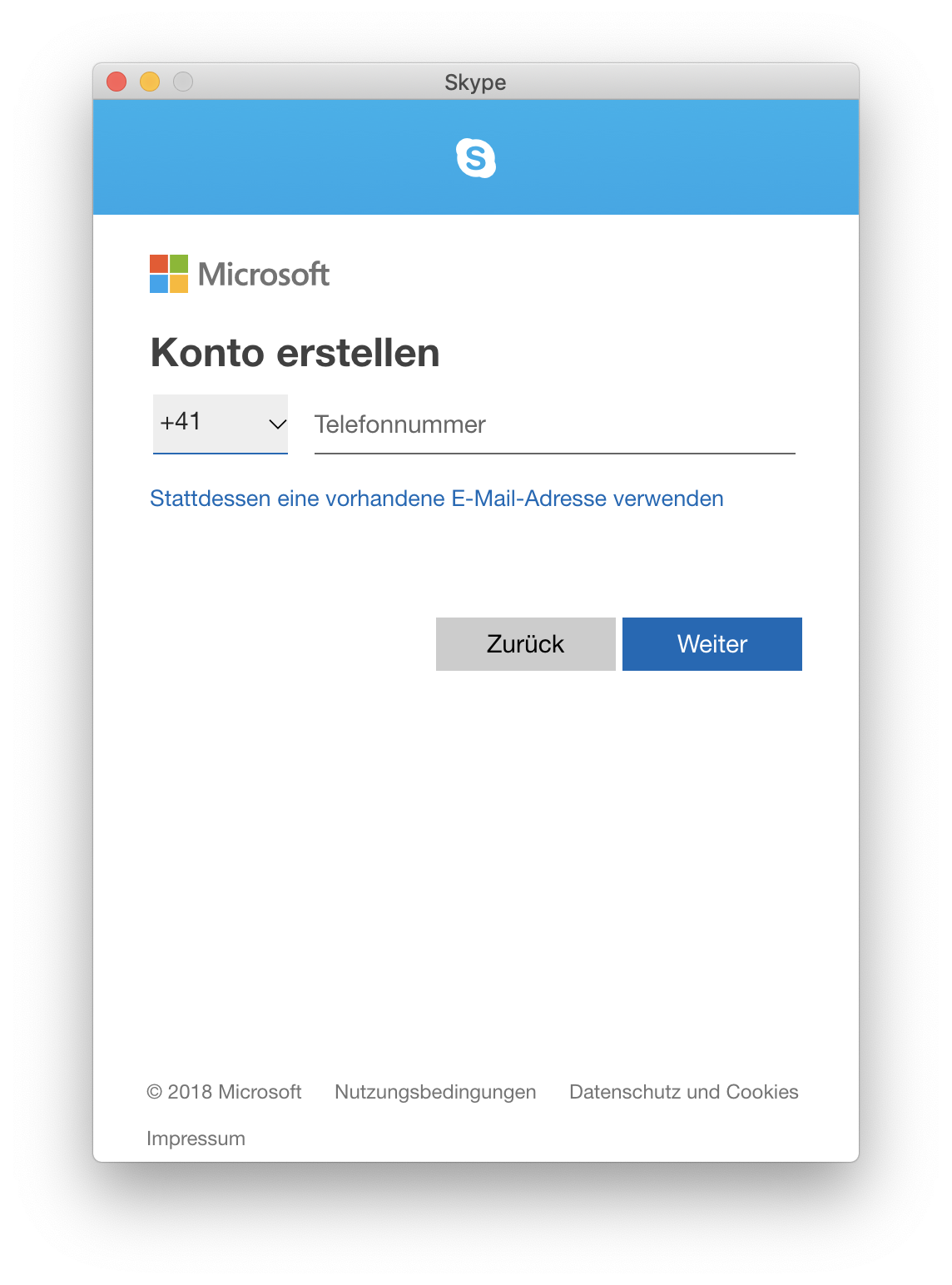 | |  | |  |
